# HTA Training for Healthcare Professionals: International Overview of Initiatives Provided by HTA Agencies and Organizations

**DOI:** 10.3389/fpubh.2022.795763

**Published:** 2022-02-10

**Authors:** Ilda Hoxhaj, Carolina Castagna, Giovanna Elisa Calabrò, Stefania Boccia

**Affiliations:** ^1^Section of Hygiene, University Department of Life Sciences and Public Health, Università Cattolica del Sacro Cuore, Rome, Italy; ^2^Department of Woman and Child Health and Public Health - Public Health Area, Fondazione Policlinico Universitario A. Gemelli IRCCS, Rome, Italy

**Keywords:** health technology assessment, training, education, healthcare professionals, HTA

## Abstract

**Background:**

Health Technology Assessment (HTA) is a multidisciplinary process that synthesizes, with a systematic, transparent, impartial and robust methodological approach, the main information on the medical, economic, ethical and social implications of the use and dissemination of a health technology. Its aim is to support decision-makers in identifying safe, effective, patient-centered and best-value health policies, in order to promote an equitable, efficient, and high-quality health system. Given the continued application of innovative technologies into clinical practice, healthcare professionals need to be able to adequately evaluate these technologies using evidence-based approaches such as HTA. Therefore, the implementation of training in HTA is crucial. The aim of this study was to investigate existing HTA training initiatives for healthcare professionals provided by international HTA agencies and organizations around the world.

**Methods:**

From March to November 2020, the websites of HTA agencies and organizations belonging to the European network for HTA (EUnetHTA) and to the International Network of Agencies for HTA (INAHTA), and the website of the HTA International (HTAi), were explored for identifying the HTA training initiatives directed to healthcare professionals. In addition, we screened the training initiatives proposed at European level by EUnetHTA as part of its Joint Actions and conducted in collaboration with its public-private partners. Specific keywords were searched in English and adapted to French, Portuguese, Spanish, Italian and German. Data extraction of the retrieved training initiatives was conducted from November 2020 to February 2021 and considered the following information: agency, country, website, coordinator, type of initiative, target, topic, main contents, and language.

**Results:**

Out of 124 agencies/organizations/EUnetHTA public-private partners screened, only 21 provided training initiatives for healthcare professionals. A total of 55 training initiatives were analyzed, 85.5% of which were delivered at the European level and 14.5% at the international level. The countries with a greater number of courses were: Austria, Argentina, Spain, Portugal, and the United Kingdom. Twenty-one training initiatives focused on HTA application and methodology while 34 on specific HTA domains, particularly on the economic one. The technologies covered were mainly drugs.

**Conclusions:**

Our study revealed a limited number of HTA training programs targeting healthcare professionals. HTA supports the decision-making processes concerning the use and application of health technologies with scientific evidence. Indeed, training of healthcare professionals in this field should be a key driver in implementing evidence-based healthcare choices and through rigorous methodological approaches such as HTA, in order to ensure proper health governance and value-based application of technological innovations in clinical practice. Therefore, capacity building of healthcare professionals in this area should be enhanced by using appropriate and effective training initiatives and educational strategies.

## Introduction

In the last 20 years, a growing development of innovative health technologies not associated with an increase in resources, has characterized health contexts around the world (1).

Health technologies include different types of interventions (e.g., drugs, devices, medical and surgical procedures, healthcare organizational, and managerial systems) and represent a major driver of costs for healthcare systems (2). Therefore, to ensure the sustainability of health systems, the interest for “disinvestment” in healthcare increased (3). Indeed, today a necessary goal for modern healthcare systems is to disinvest from low-value health technologies and to reinvest in high value ones (2) and, in order to tackle these challenges, evidence-based approaches, such as Health Technology Assessment (HTA), are needed (2). A planned and systematic evaluation of health technologies is necessary to ensure the introduction and implementation of technological innovations in different healthcare settings and at all levels of health services, in an appropriate way (4). HTA is defined as “a multidisciplinary process that uses explicit methods to determine the value of a health technology at different points in its lifecycle. The purpose is to inform decision-making in order to promote an equitable, efficient, and high-quality health system. A health technology is an intervention developed to prevent, diagnose, or treat medical conditions; promote health; provide rehabilitation; or organize healthcare delivery. The intervention can be a test, device, medicine, vaccine, procedure, program, or system” (5).

HTA plays an essential role to inform stakeholders in the planning and designing of value-based health policies, aiming to promote an equitable, efficient, and high-quality health system (5) and it is recognized internationally as a valuable tool to support policy makers in decision making (6). By maximizing the potential of HTA, policy makers would be able to implement decisions that support the benefits of technologies or interventions, recognize their value and overcome uncertainties, in order to improve population health (7).

Given the application of innovative technologies into clinical practice, healthcare professionals need to be able to properly know and assess these technologies using modern methods of analysis, such as clinical, economic, organizational, comparative effectiveness, ethical and social. To ensure that, a broad range of competencies is needed.

The skills required of healthcare professionals to use the HTA approach appropriately include a range of scientific, analytical and also organizational-managerial competencies (8).

In 2002, an European survey evaluating training and education initiatives in HTA, showed a lack of training/education in this field, in European Union (EU) member countries (9). Only a few countries (Poland, Hungary, Estonia, and Latvia) were more “active” in terms of training initiatives in HTA and many Eastern European countries expressed the need of implementing the knowledge and application of an evidence-based tool such as HTA (9).

Taking into account the scientific and technological advances achieved in the last two decades, it appears necessary that healthcare professionals are appropriately trained to know, recognize and use in a relevant way the information useful for the introduction and implementation of healthcare technologies. Therefore, the implementation of education and training in HTA is strongly recommended.

For these reasons, the aim of our study was to investigate the current state of HTA training for healthcare professionals and to map existing training courses/initiatives in HTA provided by international HTA agencies and organizations all over the world for this target population.

## Methods

From March to November 2020, the websites of HTA agencies and organizations belonging to the European network for Health Technology Assessment (EUnetHTA) and to the International Network of Agencies for HTA (INAHTA), and the website of the HTA International (HTAi, an organization representing a variety of stakeholders who have interests in HTA), were explored for identifying the HTA training initiatives directed to healthcare professionals. In addition, we screened the training initiatives proposed at European level by EUnetHTA as part of its Joint Actions (JA) and conducted in collaboration with other public-private partners such as the International Society for Pharmacoeconomics and Outcomes Research (ISPOR) and the Innovative Medicines Initiative (IMI).

### Search Strategy

Two researchers (C.C., I.H.) independently conducted the online screening process on the websites of EUnetHTA (10), INAHTA (11) and HTAi (12). On HTAi website the search was carried out only for non-profit organizations while for EUnetHTA and INAHTA the websites of all the members belonging to the two HTA networks were consulted, without restrictions on the type of entity (HTA agency, Academy, private or non-profit organization). All websites were accessed and searched entering specific keywords (“course,” “training,” “seminar,” “workshop,” “Massive Open Online Courses – MOOC,” “HTA”) in the query box. The search was conducted in English language and then adapted to French, Portuguese, Spanish, Italian, and German, in order to retrieve courses conducted at national level in different countries. Education and training initiatives on the general HTA methodology or on specific HTA domains were considered eligible for inclusion.

### Data Extraction

Data extraction was conducted from November 2020 to February 2021 independently by two researchers (I.H., C.C.) and disagreements were solved through discussion with a third researcher (G.E.C.). Only the initiatives with an available and accessible link were included.

The following data were collected for each training initiative retrieved:

i) information related to the agency providing the course: name, country, website;
ii) information related to the training initiative: title, coordinator, modalities of delivering the courses (if attendance or online course, seminar, workshop), target, topic, objectives, main contents, and language.

### Data Synthesis

The included training initiatives were grouped in the following categories:

i) training initiatives provided by European HTA agencies/organizations,
ii) training initiatives provided at EU level by public-private partners of EUnetHTA and its JA,
iii) training initiatives provided by non-EU HTA agencies/organizations.

For each category, the training initiatives were described according to the main topic covered such as principles**, **general methodology, application of HTA and specific domains of the EUnetHTA core model (13).

## Results

A total of 124 HTA agencies/organizations/EUnetHTA public-private partners were screened after removing the duplicates among EUnetHTA, INAHTA and HTAi. Table 1 shows the list. Eighteen HTA agencies/organizations–15 European and three internationals—developed training initiatives in the HTA field. Among the 15 European agencies/organizations, four were in Spain (27%), three in United Kingdom (UK) (20%) and three in Austria (20%), while of the remaining HTA agencies/organizations, five were in Portugal, Italy, the Netherlands, Belgium, and Romania (33%). Internationally, only three agencies/organizations (one in Canada, one in Australia and one in Argentina) developed training initiatives in HTA. Overall, starting from 2009 until 2021, 55 HTA training initiatives (14–67) were retrieved (Table 2), of which 54.5% (*n* = 30) provided by European HTA agencies/organizations (14–43), 14.5% (*n* = 8) by international ones (60–67) and, in addition, 31% (*n* = 17) publicly available at EUnetHTA website (27, 44–59) (Table 3).

**Table 1:** List of HTA agencies/organizations belonging to INAHTA, HTAi and EUnetHTA network, and public-private partners of EUnetHTA and its JA, which have been consulted.

**Country**	**HTA agency/organization**	**INAHTA**	**HTAi**	**EUnetHTA**
Argentina	IECS—Institute for Clinical Effectiveness and Health Policy	x		
Australia	AHTA—Adelaide Health Technology Assessment	x	x	
	ASERNIP-S—Australian Safety and Efficacy Register of New Interventional	x		
	Procedures—Surgical			
	PBAC&MSAC—Pharmaceutical Benefits Advisory Committee		x	
Austria	AIHTA—Austrian Institute for Health Technology Assessment	x		x
	UMIT—University for Health Sciences, Medical Informatics and Technology			x
	GOG—Gesundheit Österreich GmbH/Geschäftsbereich	x		x
	HVB—Hauptverband der Österreichischen Sozialversicherungsträger (Association of Austrian Social Insurance Institutions)			x
Belgium	KCE—Belgian Health Care Centre	x		x
	IPH—Scientific Institute of Public Health RIZIV—INAMI- Rijksinstituut voor Ziekte- en Invaliditeitsverzekering			x x
Brazil	ANS—National Regulatory Agency for Private Health Insurance and Plans	x		
	CONITEC—National Committee for Technology Incorporation	x	x	
	MoH—Ministry of Health of Brazil		x	
Bulgaria	NCPHA—National Center of Public Health and Analyses			x
Canada	CADTH—Canadian Agency for Drugs and Technologies in Health	x	x	
	IHE—Institute of Health Economics	x	x	
	INESS—Institut national d'excellence en santé et en services sociaux^*^	x		
	OH—Ontario Health	x		
China	CDE—Center for Drug Evaluation, Taiwan	x		
Colombia	IETS—Instituto de Evaluación Tecnológica en Salud	x		
Croatia	MIZ—Ministry of Health of the Republic of Croatia			x
	CHIF—Croatian Health Insurance Fund			x
	CIPH—Croatian Institute of Public Health			x
Cyprus	MoH Cyprus—Ministry of Health of Cyprus			x
Czech Republic	MoH Czech—Ministry of Health of the Czech Republic			x
	SUKL—State Institute for Drug Control			x
Denmark	DEFACTUM (formerly CFK)	x		x
Estonia	UTA—Institute of Family Medicine and Public Health			x
Finland	FinCCHTA—Finnish Coordinating Center for Health Technology Assessment	x	x	x
	FIMEA—Finnish Medicines Agency			x
	THL—National Institute for Health and Welfare			x
France	HAS—French National Authority for Health (Haute Autorité de Santé)	x	x	x
	AP-HP—Assistance publique- Hopitaux de Paris, FRANCE	x		
Germany	DIMDI—German Institute for Medical Documentation and Information			x
	GBA—Gemeinsamer Bundesausschuss	x		x
	IQWIG—Institute for Quality and Efficiency in Health Care		x	x
Greece	EKAPTY-NKUA—National and Kapodistrian University of Athens			x
	EKAPTY SA—National Evalution Center of Quality and Technology in S.A.-			x
	EOF—National Organization for Medicines			x
	EOPYY—National Organisation for Healthcare Provision			x
	IFET—Institute of Pharmaceutical Research and Technology			x
	OCSC—Onassis Cardiac Surgery Centre			x
Hungary	NIPN—National Institute of Pharmacy and Nutrition			x
	SU—Health Services Management Training Center			x
Indonesia	CEEBM Center for Clinical Epidemiology-Evidence Based Medicine at Cipto Mangunkusumo Hospital		x	
Ireland	HIQA—Health Information and Quality Authority	x	x	x
	NCPE—National Centre for Pharmacoeconomics, St. James Hospital			x
Italy	AGENAS—National Agency for Regional Health Services	x		x
	UCSC Gemelli- University Hospital A. Gemelli		x	x
	AIFA—Italian Medicines Agency	x		x
	CRUF/AOUIVR—Centro Regionale Unico sul Farmacia del Veneto			x
	DGFDM IT—Sede del Ministro–Ministero della salute			x
	RER—Regione Emilia-Romagna			x
	UVTA/AOP—Unita di Valutazione Technology Assessment			x
	Veneto/CRUF—Regione Del Veneto–Area Sanità e Sociale			x
Kazakhstan	SK-NRCHD—Salidat Kairbekova National Research Center for Health Development^*^	x		
Korea	NECA—National Evidence-based healthcare Collaborating Agency^*^	x	x	
Latvia	NVD—National Health Service			x
Lithuania	HI—The Institute of Hygiene			x
	VASPVT—State Health Care Accreditation Agency			x
	VVKT—State Medicines Control Agency of Lithuania			x
Malaysia	MaHTAS—Health Technology Assessment Section, Ministry of Health Malaysia^*^	x	x	
Malta	DPA/MoH Malta—Directorate for Pharmaceutical Affairs			x
Netherlands	EUR—Erasmus Universiteit Rotterdam			x
	UU—Utrecht University			x
	ZIN—National Health Care Institute	x	x	x
	ZonMw—The Netherlands Organisation for Health Research and Development^*^	x		
Norway	NIPH—Norwegian Institute of Public Health	x		
	HDIR—Norwegian Directorate of Health			x
	NIPHNO (formerly NOKC) —The Norwegian Institute of Public Health		x	x
	NOMA—Norwegian Medicines Agency			x
	Norwegian Centre for E-health Research		x	
Peru	IETSI—Institute of Health Technology Assessment and Research	x		
Poland	AOTMiT—Agency for Health Technology Assessment and Tariff System	x		x
Portugal	ACSS IP—Administração Central do Sistema de Saúde, I.P.			x
	INFARMED—National Authority of Medicines and Health Products			x
Romania	NIPHB—Institutu National De Sanatate Publica (INSP)			x
	NSPHMPDB—National School of Public Health, Management and Professional			x
	Development			
	UBB—Babes-bolayi University, Cluj School of Public Health			x
Russian Federation	CHQA—Center for Healthcare Quality Assessment and Control	x		
	HTA Association		x	
Singapore	ACE—Agency for Care Effectiveness	x		
Slovakia	MoH Slovak Republic—Ministry of Health of the Slovak Republic			x
	UniBA FOF—Comenius University in Bratislava			x
Slovenia	JAZMP—Public Agency of the Republic of Slovenia for Medicinal Products and			x
	Medical Devices			
	MoH Slovenia—Ministry of Health of the Republic of Slovenia			x
	NIJZ—National institute of Public Health (NIJZ)			x
Spain	AEMPS—Agencia Española de Medicamentos y Productos Sanitarios			x
	AETS-ISCIII—The Instituto De Salud Carlos III			x
	AETSA—Andalusian HTA Agency	x	x	x
	AquAS—Agency for Health Quality and Assessment of Catalonia	x		x
	AVALIA FNS—Fundacion Profesor Novoa Santos AVALIA-T—Galician Agency for HTA	x	x	x x
	BIOEF—Basque Foundation for Health Innovation and Research			x
	DGFPS MSPSI-Directorate General for Pharmacy and Health Care Products			x
	FPS—Fundación Pública Andaluza Progreso y Salud			x
	FUNCANIS—Fundación Canaria de Investigación Sanitaria			x
	IACS—Health Sciences Institute in Aragon, SPAIN	x		
	OSTEBA—Basque Office for Health Technology Assessment- Ministry for Health	x	x	x
	SESCS—Evaluation AND Planning Unit–Directorate of the Canary Islands Health Service			x
Sweden	SBU—Swedish Agency for Health Technology Assessment and Assessment of	x		x
	Social Services			
	MPA—Medical Products Agency			x
	TLV—Dental and Pharmaceutical Benefits Agency		x	x
Switzerland	SNHTA—Swiss Network for HTA			x
	SFOPH—Swiss Federal Office of Public Health^*^	x	x	
Tunisia	INEAS—National Authority for Assessment and Accreditation in Healthcare^*^	x	x	
Ukraine	MoH Ukraine—HTA Department of SEC of Ministry of Health of Ukraine^*^	x		x
United Kingdom	HTW—Health Technology Wales	x	x	
	HIS—Healthcare Improvement Scotland	x	x	x
	NICE—National Institute for Health and Care Excellence	x	x	x
	NIHR—National Institute for Health Research^*^	x		
	AWTTC—All Wales Therapeutics and Toxicology Centre	x	x	x
United States	AHRQ—Agency for Healthcare Research and Quality	x	x	
	Blue Cross Blue Shield Association		x	
	CMTP—Center for Medical Technology Policy			
	ICER—Institute for Clinical and Economic Review		x	
	Kaiser Permanente		x	
	PCORI—Patient-Centered Outcomes Research Institute (USA)		x	
Uruguay	HAD—Health Assessment Division, Ministry of Public Health	x		
	HAD—Health Assessment Division, Ministry of Public Health	x		
	EUnetHTA JA2			x
**Joint Action (JA) and public-private partners of EUnetHTA**				
European Union	IMI—Innovative Medicines Initiative			x
	ISPOR—International Society for Pharmacoeconomics and Outcomes Research			x

* *Websites not available*.

**Table 2:** Number of HTA training initiatives by year, country, and promoting agency/organization.

**Year**	**N. of HTA training initiatives**	**Country**	**Agency/organization (N. of initiatives)**
2021	3	Austria	UMIT (n. 3)
2020	25	Portugal	INFARMED (n. 2)
		Spain	AEMPS (n. 1)
		UK	HTW (n. 1)
		Australia	AHTA (n.1)
		Argentina	IECS (n. 6)
		EU	ISPOR^*^ (n. 13)
		EU	IMI (n. 1)
2019	7	Italy	UCSC Gemelli (n. 1)
		Portugal	INFARMED (n. 2)
		Spain	FIISC (n. 1)
		UK	AWTTC (n. 1)
		UK	HTW (n. 1)
		EU	ISPOR^*^ (n. 1)
2018	3	Austria	AIHTA (n. 1)
		Austria	GOG (n. 1)
		EU	ISPOR^*^ (n. 1)
2017	3	Austria	AIHTA (n. 1)
		Austria	GOG (n. 1)
		EU	ISPOR^*^ (n.1)
2016	7	Austria	GOG (n. 1)
		Italy	UCSC Gemelli (n. 1)
		Portugal	INFARMED (n. 1)
		Spain	AEMPS (n. 1)
		Spain	AETSA (n. 1)
		EU	ISPOR^*^ (n. 1)
		EU	EUnetHTA JA2 (n. 1)
2015	3	Canada	CADTH (n.1)
		EU	ISPOR^*^ (n. 1)
		EU	EUnetHTA JA2 (n. 1)
2014	1	EU	EUnetHTA JA2 (n. 1)
2013	0	-	-
2012	1	Spain	BIOEF (n. 1)
2011	0	-	-
2010	1	Belgium	KCE (n. 1)
2009	2	Austria	AIHTA (n. 1)
		Spain	AEMPS (n. 1)

* *One training initiative provided by ISPOR was replicated annually for 6 years (2015–2020)*.

**Table 3:** Number of overall training initiatives provided by EU and non-EU HTA agencies/organizations at national/international level.

**Country**	**Agency/organization**	**N. of HTA training initiatives**
Argentina	IECS	6
Australia	AHTA	1
Austria	AIHTA	3
	UMIT	3
	GOG	3
Belgium	KCE	1
Canada	CADTH	1
Italy	UCSC Gemelli	2
The Netherlands	Erasmus Universiteit Rotterdam	1
Portugal	INFARMED	5
Romania	NSPHMPDB	1
Spain	AEMPS	3
	FIISC	1
	BIOEF	1
	AETSA	1
United Kingdom	NICE	1
	AWTTC	2
	HTW	2
Public-private partners of EUnetHTA and its JA	ISPOR	13
	EUnetHTA JA2	3
	IMI	1

**Figure 1:**
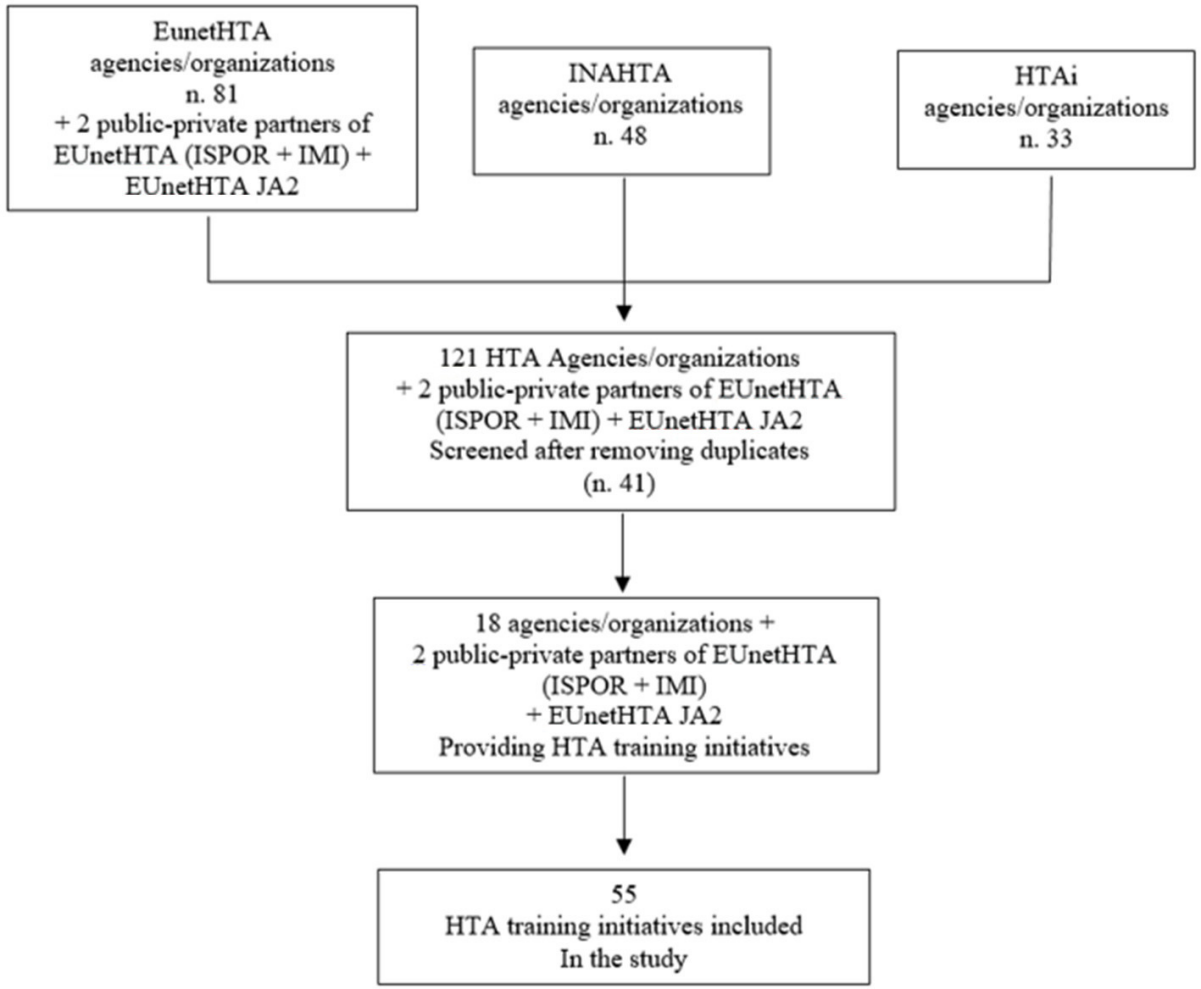
Flowchart of the selection process.

The screening process is shown in Figure 1.

Overall, Austria (*n* = 9), Argentina (*n* = 6), Spain (*n* = 6), Portugal (*n* = 5), and UK (*n* = 5) were the countries with the majority of training initiatives.

Regarding the topic of the training initiatives included in our study, 21 of them (14, 16, 18, 23–26, 31, 32, 36, 37, 39, 43, 56–61, 66, 68) addressed the general methodology and applications of HTA, and 34 focused on specific HTA domains, especially on economic evaluations (*n* = 22). The technologies covered were the following: health technologies in general (*n* = 42), drugs (*n* = 12), genetic therapies (*n* = 2), and medical devices (*n* = 1).

### Training Initiatives Provided by European HTA Agencies/Organizations

Table 4 shows the training initiatives provided by European HTA agencies/organizations at national level.

**Table 4:** Training initiatives provided by EU HTA agencies/organizations at national level.

**Country**	**Promoting agency/organization**	**Training initiatives title**	**Year of delivery**	**Type of training initiative**	**Target**	**Topic**	**Technology addressed in the course**	**Language**
Austria	AIHTA—Austrian Institute for Health Technology Assessment	1st Workshop of the EUnetHTA Task Force on HTA and Medical Devices (14)	2018	Workshop	HTA professionals	HTA regulation and legal framework	Medical devices	English
		Workshop series: Ethik and HTA (15)	2017	Workshop	HTA professionals, health policy decision-makers, experts in applied ethics in HTA	Ethical methodology in HTA	Health technology	German
		Workshop Health Technology Assessment (16)	2009	Workshop	Healthcare decision makers	HTA methodology and application	Health technology	English/German
	UMIT— University for Health Sciences, Medical Informatics and Technology	Modeling Approaches for HTA: A Practical Hands-on Workshop (17)	2021	Three-day course	Healthcare and health policy organizations, national HTA Agencies; Pharmaceutical and medical device industry; Academia and research institutions; Health insurances/sickness funds; Consultancy organizations.	HTA methodology	Health technology	English
		Introduction to health technology assessment HTADS—program on health technology (1)assessment and decision sciences (18)	2021	Four-day course	Healthcare and health policy organizations, national HTA Agencies; Pharmaceutical and medical device industry -Academia and research institutions -Health insurances/sickness funds-Consultancy organizations	HTA methodology	Health technology	English
		Causal inference for assessing effectiveness in real world data and clinical trials: a practical hands-on workshop (19)	2021	Five-day course	Healthcare and health policy organizations, national HTA agencies regulatory agencies; pharmaceutical and medical device industry, academia and research institutions, health insurances, consultancy organizations.	Clinical utility (effectiveness)	Health technology	English
	GOG—Gesundheit Österreich GmbH/Geschäftsbereich	3rd summer school pharmaceutical pricing and reimbursement policies (20)	2018	Summer school/5 days training course	Professionals of public and non-for profit institutions working in the field of pricing and reimbursement of medicines	Economic evaluation	Drugs	English
		2nd summer school pharmaceutical pricing and reimbursement policies (21)	2017	Summer school/5 days training course	Professionals of public and non-for profit institutions working in the field of pricing and reimbursement of medicines	Economic evaluation	Drugs	English
		1st summer school pharmaceutical pricing and reimbursement policies (22)	2016	Summer school/5 days training course	Professionals of public and non-for profit institutions working in the field of pricing and reimbursement of medicines	Economic evaluation	Drugs	English, and simultaneous translation into Russian
Belgium	KCE— belgian health care centre	HTA workshop | collaboration between HTA agencies in practice: learning from actual experiences (23)	2010	Workshop	HTA agencies	HTA methodology	Health technology	English
Italy	UCSC Gemelli	Health impact assessment e health technology assessment (24)	2019	In attendance course	Researchers and health managers	HTA and HIA methodology	Health technology	Italian
		Health technology assessment (25)	2016	In attendance course	Healthcare professionals	HTA methodology and application	Health technology	Italian
Netherlands	Erasmus Universiteit Rotterdam	MOOC health technology assessment (26)	N.A.^*^	Massive open online course (MOOC)	Health economics policy and law master students, health economics master students and the research master students	HTA methodology and application	Health technology	English
Portugal	INFARMED- National Authority of Medicines and Health Products	Health technology assessment training program (68)	2016	Online course	Professionals from government and health insurance funds, HTA bodies, public and private payers and health plans, industry, academia, and patient group representatives;	HTA methodology and application	Health technology	English
		Pharmacovigilance course (Curso de atualização em farmacovigilância) (28)	2020	Online course	Health professionals	Clinical utility (safety)	Drugs	Portuguese
		Curso Pós-Graduado De Atualização: Assessing Therapeutic Efectiveness In Drug Lifecycle (29)	2019	In attendance course	Academics and health care professionals, PhD students and master students	Clinical utility (effectiveness)	Drugs	English
		Artificial intelligence in health: governance, accountability and decision-making (Inteligência artificial em saúde: governança, responsabilidade e tomada de decisão) (30)	2019	In attendance course	Higher education professionals and students in the fields of health, social sciences, management, computer science and engineering.	Ethical and legal aspects	Digital health technology	Portuguese
		Introduction to value-based health care management (Introdução à Gestão de Cuidados de Saúde Baseada em Valor) (31)	2020	b-learning course	Healthcare professionals	HTA application and Value Based Healthcare	Health technology	English
Romania	NSPHMPDB- The National School of Public Health, Management and Professional Development	Training programs in both public health and management (32)	N.A.^*^	In attendance Courses	All categories of staff in public health and management and other areas of the health system	HTA methodology and application	Health technology	Romanian
Spain	AEMPS- La Agencia Española de Medicamentos y Productos Sanitarios	Technical training of biosimilar medicines (Capacitación técnica de medicamentos biosimilares) (33)	2020	Online course	Specialized and qualified representatives from seventeen Ibero-American countries.	Legal and regulatory processes	Drugs	Spanish
		3rd training course in standards of good clinical practice for independent researchers (3° curso de formación en normas de buena práctica clínica para investigadores independientes) (34)	2009	In attendance course	Clinical researchers	Ethical and legal aspects	Health technology and drugs	Spanish
		Practical course for conducting pharmacoepideomyology studies with the bifap database (curso practico para la realización de estudios de farmacoepidemiologia con la base de datos bifap) (35)	2016	In attendance course	Public researchers	Clinical utility (safety)	Drugs	Spanish
	FIISC—Funcanis_Fundación Canaria de Investigación Sanitaria	Application of molecular biology in the diagnosis and follow-up of chronic lymphocytic leukemia (Aplicación de la Biología molecular en el diagnóstico y seguimiento de la Leucemia Linfocítica crónica) (36)	2019	In attendance Course	Graduates and certified lab technicians	HTA application	Genetics, molecular biology and mass sequencing	Spanish
	BIOEF— Basque Foundation for Health Innovation and Research	UPV/EHU summer course on health research and innovation (37)	2012	In attendance course	Healthcare professionals, clinical researchers	HTA applications	Health technology	Spanish
	AETSA— Andalusian HTA Agency	Introduction of the economic assessment in the health technology assessment (38)	2016	Workshop	Andalusian public health system (SSPA) professionals interested in financial evaluation	Economic evaluation	Health technology and drugs	Spanish
United Kingdom	NICE	Seminars (39)	N.A.^*^	Seminars; Advanced workshops	Pharmaceutical, medical technology or cell and gene therapy sectors	HTA applications	Health technology, drugs, gene therapy	English
	AWTTC— All Wales Therapeutics and Toxicology Centre	AWMSG training day (40)	2019	In attendance course	Members and deputies of AWMSG; new medicines group and the all wales prescribing advisory group; medicines and therapeutics committees	Economic evaluation	Orphan medicines	English
		Adverse drug reactions: reporting makes medicines safer (41)	N.A.^*^	Online course	Healthcare professionals	Clinical utility (Safety)	Drugs	English
	HTW— Health Technology Wales	Health technology assessment and economics (42)	2019	Workshop	NHS front line staff; NHS Financial, Medical and Planning Directors; Care commissioners; Workforce managers; Patients; Members of the public; Academia; Technology developers; Industry representatives	Economic evaluation	Health technology	English
		Value in health week (43)	2020	Webinar	All stakeholders	HTA dimensions and applications	Health technology	English

* *The year in which training initiative was provided was Not Available (N.A.)*.

#### Training Initiatives Addressing Principles, General Methodology, and Application of HTA

Among the 30 training initiatives (14–43) provided by European HTA agencies/organizations, 47% (*n* = 14) addressed the general aspects of HTA principles, methodology and application: Austria (*n* = 3); Spain (*n* = 2); Portugal (*n* = 2); UK (*n* = 2); Italy (*n* = 2); Romania (*n* = 1); Belgium (*n* = 1) and the Netherlands (*n* = 1).

The Austrian Institute for Health Technology Assessment GmbH (AITHA) organized in 2009 a workshop in English and German for healthcare decision-makers, addressing HTA application and methodology, such as systematic review research, medical statistics and clinical epidemiology appraisal, and decisions in health policy (16). These topics were covered also in two English courses organized in 2021, by The University for Health Sciences, Medical Informatics and Technology (UMIT) in Austria, directed to healthcare professionals and health policy organizations, national HTA agencies, industry, academia and research institution and consultancy organizations (17, 18).

In Spain, a course offered by the Fundación Canaria de Investigación Sanitaria for lab technicians, discussed the HTA applications in genetics and molecular biology, technologies used and mass sequencing (36). In 2016, the course by the Basque Foundation for Health Innovation and Research (BIOEF), for healthcare professionals and clinical researchers, covered HTA applications and the translation from research to clinical practice (37).

Two courses (27, 31) were organized by the National Authority of Medicines and Health Products (INFARMED) in Portugal. The 2016 English course, on HTA principles and multi-perspective approach, was directed to professionals from all relevant sectors, including government and health insurance funds, HTA bodies, public and private payers and industry, academia, and patient group representatives (27). In 2020, a b-learning English course focused on HTA application and Value Based Health care was directed to healthcare professionals (31). The latter were also the topic of a webinar organized in October 2020, by the Health Technology Wales, considering the benefits and challenges of their application in Wales' national health system (43). In UK, HTA principles and application regarding health technology, drugs and gene therapy were part of the NICE seminars/workshops for professionals in the pharmaceutical, medical technology or gene therapy sectors (39).

In Italy, in-attendance courses regarding the HTA methodology in Italian language were provided by Università Cattolica del Sacro Cuore, Policlinico Universitario Agostino Gemelli IRCCS (Rome) for healthcare professionals in 2016 (25) and for researchers and health managers in 2019 (24).

Also, in Romania the National School of Public Health, Management and Professional Development offers periodically several courses in this topic for professionals in public health and management sectors (32). The Belgian Health Care Knowledge Centre (KCE) organized in 2010 a workshop in English addressing professionals working on HTA agencies on HTA applications, collaboration and cooperation (23). In the Netherland, the Erasmus University Rotterdam launched a MOOC in English on the HTA principles and their application in the policy context about new and existing health technologies (26).

#### Training Initiatives Addressing HTA Specific Domains of the EUnetHTA Core Model

Among the 30 training initiatives provided by European HTA agencies/organizations, 16 (53%) focused on specific HTA domains, as following: 6 initiatives on economic evaluations (20–22, 38, 40, 42); 5 on ethical and legal aspects (14, 15, 30, 33, 34), two on clinical effectiveness (19, 29) and three courses on safety (28, 35, 41).

##### Economic Evaluations

Health economic evaluation was the topic of 6 courses (20–22, 38, 40, 42): three in Austria (20–22), two in UK (40, 42) and one in Spain (38). The Austrian National Public Health Institute (GOG) organized, for 3 years (2016–2018), a 5-day training course on pharmaceutical pricing and reimbursement policies for professionals of public and non-for profit institutions working in this field (20–22).

In UK, the All Wales Therapeutics and Toxicology Centre agency organized in 2019 an in-attendance course on the health opportunity costs of orphan medicines and the policy implications of decision-making (40). Instead, in October 2020, the Health Technology Wales offered to all stakeholders a webinar on economic evaluation methodology used to understand the cost effectiveness of healthcare technologies and their impact on resources (42).

In Spain, a workshop in Spanish organized by the Andalusian HTA Agency (AETSA) for professionals of the Andalusian public health system was held in 2016 (38). The course topic was the importance and typology of economic evaluation for health technologies and pharmaceuticals.

##### Ethical and Legal Aspects

Five courses (14, 15, 30, 33, 34) addressed the ethical and legal aspects of health technologies: two courses in Austria (14, 15), two in Spain (33, 34) and one in Portugal (30). In Austria, AIHTA organized a course in German language in 2017 regarding the ethical methodology, targeted for HTA professionals, health policy decision-makers and experts in applied ethics in HTA (15). The other workshop, addressing only HTA professionals, was held in 2018, in English, on HTA regulatory and legal frameworks for medical devices (14). In Spain, the Agencia Española de Medicamentos y Productos Sanitarios (AEMPS), organized in attendance courses in Spanish, for clinical researchers in 2009 (34) and for public researchers in 2020 (33), on the ethical aspects, legal and regulatory processes of drug administration. The ethical and legal implications of the use of digital technologies in healthcare were covered at the Portuguese in-attendance course offered by INFRAMED, addressed to higher education professionals in the fields of health, social sciences, management, computer science and engineering (30).

##### Clinical Effectiveness

Two courses focused on the effectiveness of health technologies (19, 29). In Portugal, INFRAMED organized in 2019 an in-attendance English course on the therapeutic effectiveness and the evaluation methodology, directed to academic and healthcare professionals (29). In Austria, UMIT provided in 2021 an English course on the methodology for assessing the effectiveness of real world data and clinical trials, to healthcare professionals in policy organizational, national HTA agencies, health industry or academia (19).

##### Safety

Three courses addressed the safety of health technologies: one course in Portugal (28), one in UK (41) and one in Spain (35). In Portugal, INFARMED provided in 2020 an online course in Portuguese for healthcare professionals on HTA application in pharmacovigilance (28). Pharmacovigilance was the topic also two other courses: an online course organized by the All Wales Therapeutics and Toxicology Centre in UK (41), and an in-attendance course on 2016 by AEPMS in Spain (35).

#### Training Initiatives Provided at EU Level by Public-Private Partners of EUnetHTA and Its JA

We screened also the training initiatives proposed at European level by EUnetHTA as part of its JA and conducted in collaboration with other public-private partners such as ISPOR and IMI.

Seventeen training initiatives were publicly available at EUnetHTA website (27, 44–59), of which 13 were organized by ISPOR (27, 45–55), three by EUnetHTA JA2 (56–58) and one by IMI (59). These initiatives are reported in Table 5.

**Table 5:** Training initiatives^*^ provided at EU level by public-private partners of EUnetHTA.

**Promoter**	**Training initiatives title**	**Year of delivery**	**Type of training initiative**	**Target**	**Topic**	**Technology addressed in the course**
ISPOR— International Society for Pharmacoeconomics and Outcomes Research	Health technology assessment training program (27)	2015; 2016; 2017; 2018; 2019; 2020	In attendance course	Users and doers in government - Public and private payers industry, health plans, academia - Patient group representatives	Economic evaluation (budget impact analysis; cost evaluation)	Health technology
	Modeling health care costs- part I characteristics of health care costs (44)	2020	Virtual training	Healthcare stakeholders	Economic evaluation (budget impact analysis; cost evaluation)	Health technology
	Modeling health care costs—Part II: methods and guidelines for estimating health care costs (45)	2020	Virtual training	Healthcare stakeholders	Economic evaluation (cost-effectiveness analysis; cost-utility analysis)	Health technology
	Modeling health care costs—part III: estimation from censored data (46)	2020	Virtual training	Healthcare stakeholders	Economic evaluation (methodology; budget impact analysis)	Health technology
	Markov model toolkit: concepts, assumptions and examples (47)	2020	Virtual training	Healthcare stakeholders	Economic evaluation (Markov modelling)	Health technology
	Introduction to pharmaco-economics (48)	2020	Virtual training	Healthcare stakeholders	Economic evaluation (cost-effectiveness analyses)	Drugs
	Cost-of-illness/cost-estimation (COI/CE) (49)	2020	Virtual training	Healthcare stakeholders	Economic evaluation (cost-of-illness/cost-estimation)	Health technology
	Cost-minimization/cost-consequence (CMA/CCA) (50)	2020	Virtual training	Healthcare stakeholders	Economic evaluation (cost-of-illness /cost-estimation)	Health technology
	Introduction to budget impact analysis (BIA) - part I (51)	2020	Virtual training	Healthcare stakeholders	Economic evaluation (budget impact analysis)	Health technology
	Introduction to budget impact analysis (BIA) - part II (52)	2020	Virtual training	Healthcare stakeholders	Economic evaluation (budget impact analysis)	Health technology
	Cost-effectiveness analysis (CEA) and cost-utility analysis (CUA) (53)	2020	Virtual training	Healthcare stakeholders	Economic evaluation (cost-effectiveness analysis and cost-utility analysis)	Health technology
	Patient reported outcomes: analysis and interpretation (54)	2020	Virtual training	Healthcare stakeholders	Patients reported outcomes	Health technology
	Patient reported outcomes: instrument development (55)	2020	Virtual training	Healthcare providers	Patients reported outcomes	Health technology
EUnetHTA JA2	HTA core model training course (56)	2015	In-attendance Training Course	HTA Agencies	HTA methodology (HTA core model) and application	Health technology
	Overview of HTA core model training materials (57)	2016	b-learning course	EUnetHTA members Agencies	HTA methodology (HTA core model) and application	Health technology
	Key principles of HTA or what is meant for HTA (58)	2014	Training Course	EUnetHTA stakeholders	HTA methodology and application	Health technology
IMI—Innovative Medicines Initiative	Real-world evidence in medicine development, - getreal (59)	2020	Virtual course	Pharmaceutical companies regulatory authorities health technology assessment bodies patients' organizations	HTA methodology and application	Health technology

* *All courses were in English language*.

Four training initiatives addressed HTA methodology and application (56–59), whereas 13 focused on specific domains, such as economic evaluations (*n* = 11) (27, 44–53) and Patients Reported Outcomes (PROs) (*n* = 2) (54, 55).

Among the initiatives on **HTA methodology and application**, three were organized by EUnetHTA JA2 in English and directed to stakeholders of EUnetHTA agency members (56–58). The first was held in 2014, about the key principles, definition, purpose, history and use of HTA (58). The other two, in 2015 (56) and 2016 (57), focused on HTA core model and applications to produce core HTA information. Moreover, IMI organized the course “Get Real” on the techniques, opportunities and challenges for the use of real-world evidence in medicine development, directed to healthcare professionals in pharmaceutical companies, regulatory authorities, HTA bodies, patients' organizations, consultancy companies, and academia (59).

**Training initiatives on specific HTA domains** were organized by ISPOR in English and for healthcare stakeholders. Of these 11 courses were on economic evaluations (*n* = 11) (27, 44–53) and focused on the methodology of cost-effectiveness, cost-utility and budget impact analysis. Two courses were on PROs (*n* = 2) and addressed the interpretation, analysis and the methodological issues related to the use of PRO tools (54, 55).

#### Training Initiatives Provided by Non-EU HTA Agencies/Organizations

Eight training initiatives were provided by HTA agencies/organizations at non-European level (60–67): 6 courses from an HTA organization in Argentina (62–67), one in Australia (60) and the other one in Canada (61) (Table 6). Among those, three focused on the HTA principles and methodology, whereas five on the economic evaluations.

**Table 6:** Training initiatives provided by non- EU HTA agencies/organizations at international level.

**Country**	**Promoting agency/organization**	**Training initiatives title**	**Year of delivery**	**Type of training initiative**	**Target**	**Topic**	**Technology addressed in the course**	**Language**
Australia	AHTA— Adelaide Health Technology Assessment	Health technology assessment 2020 online course handbook (60)	2020	Online course handbook	Health professionals	HTA methodology	Health technology	English
Canada	CADTH—Canadian Agency for Drugs and Technologies in Health	Health technology assessment for decision makers HTA institute 2015 (61)	2015	Three days intensive course	Decision-makers	HTA methodology	Health technology	English
Argentina	IECS— Institute for clinical effectiveness and health policy	Introducción a las Evaluaciones de Tecnologías Sanitarias y Evaluaciones Económicas (62)	2020	Distance learning course	Health professionals working in ministries, secretariats, regulatory agencies, medical directorates, managers of private health systems, pharmaceutical companies and producers of health technologies.	Economic evaluation (cost-effectiveness; cost-utility)	Health technology	Spanish
		Evaluaciones Económicas: Programación, análisis e interpretación de modelos de decision (63)	2020	Distance learning course	Health professionals working in ministries, secretariats, regulatory agencies, medical directorates, managers of private health systems, pharmaceutical companies and producers of health technologies.	Economic evaluation (cost-effectiveness; cost-utility)	Health technology	Spanish
		Estimación de costos para las evaluaciones económicas de programas, servicios y tecnologías en salud (64)	2020	Distance learning course	Health professionals working in ministries, secretariats, regulatory agencies, medical directorates, managers of private health systems, pharmaceutical companies and producers of health technologies.	Economic evaluation (cost-analysis)	Health technology	Spanish
		Diseño, programación y análisis de modelos de Markov (65)	2020	Distance learning course	Health professionals working in ministries, secretariats, regulatory agencies, medical directorates, managers of private health systems, pharmaceutical companies and producers of health technologies.	Economic evaluation (cost-effectiveness; markov model)	Health technology	Spanish
		Desarrollo e implementación de evaluaciones de tecnologías sanitarias (66)	2020	Distance learning course	Health professionals working in ministries, secretariats, regulatory agencies, medical directorates, managers of private health systems, pharmaceutical companies and producers of health technologies.	HTA methodology	Health technology	Spanish
		Análisis de impacto presupuestario (AIP) en salud (67)	2020	Distance learning course	Health professionals working in ministries, secretariats, regulatory agencies, medical directorates, managers of private health systems, pharmaceutical companies and producers of health technologies.	Economic evaluation	Health technology	Spanish

The initiatives on HTA principles and methodology were organized by the Canadian Agency for Drugs and Technologies in Health (CADTH) (61), the Australian Hand Therapy Association (AHTA) (60) and the Institute for Clinical Effectiveness and Health Policy (IECS) (66). In Canada, CADTH organized, in 2015, a 3 days intensive course on to decision-makers (61), whereas in Australia, AHTA offered in 2020 an online course directed to health professionals (60). IECS organized, in 2020 in Argentina, a distance learning course in Spanish, on the tools and knowledge necessary to apply HTA to health decision-making, for health professionals in ministries, regulatory agencies, medical directorates, private health systems and pharmaceutical companies (66). Moreover, IECS provided five distance learning courses, in 2020, targeting the same health professionals, covered the concepts of economic evaluations, such as cost-effectiveness, cost-utility, cost-minimization, and their application (62–65, 67).

## Discussion

Our study presents an overview of HTA training initiatives provided to healthcare professionals by international HTA agencies and organizations. The results of our screening showed that to date only a part of the international HTA agencies/organizations carry out training on HTA for healthcare professionals. In fact, out of 124 agencies/organizations consulted only 18 delivered specific training initiatives on HTA for our target population in recent years. Starting from 2009, until 2021, we identified only 55 publicly available courses, the majority of which organized in the last 5 years. Most of the courses were offered by European HTA agencies/organizations, particularly in Austria, Spain, Portugal and UK. Overall, the economic evaluations and general HTA methodology were the main focus of the identified training initiatives.

The first courses were organized in the period 2009–2012, by European agencies/organizations in Austria, Belgium, and Spain, on HTA methodology and ethical principles. This could be related to the EUnetHTA publication, in 2008, of the Handbook on HTA Capacity Building (69). This document highlighted the need to train the internal staff of HTA organizations, through effective educational tools, in accordance with organization and staff qualifications (69). As proposed by EUnetHTA in its handbook, training in HTA should focus on two main aspects: (1) understanding the results of HTA and implementing them in evidence-based health policies and (2) providing continuing training in HTA for its greater application in the healthcare.

National HTA capacity building was one of the focal points of the EUnetHTA JA2 (70), which since 2014, organized training courses on HTA methodology and its key concepts, for 3 consecutive years. In this regard, training of potential HTA agency members, as the practical users, improves general understanding of the HTA impact in decision-making and strengthens the practical application of tools and approaches for a sustainable cross-border HTA collaboration.

Following such initiatives, to address the probable shortage of HTA specialists in front of the large number of new and existing technologies, other European and non-European HTA agencies organized courses focusing on HTA procedures and methodologies. The HTA approach varied among counties according to national healthcare system, organization (central vs. regional); funding, insurance and reimbursement schemes (tax-based vs. social insurance-based); or the perspective used in HTA (health system vs. societal) (71).

Probably due to the fact that HTA is not mandatory in the decision-making process of health policy (69), its application in different countries is very heterogeneous, leading to a different prioritization at a global level of health technologies to bring into market. In fact, the national socio-economic perspective is one of the main drivers of decision-making, and decision-makers mostly prioritize cost-effectiveness analysis and clinical utility when introducing a new technology (72). However, the decision-making process, to be effective, should be based on multi-stakeholder cooperation and should encompass all dimensions of health technology evaluation such as medical, economics, social, legal and ethical (5). The results of our study show that organizational, ethical, social and legal aspects are less addressed in training courses than the economic domain, confirming also in the training field a greater interest in the economic aspects related to health technologies to be evaluated. However, training on the general HTA methodology is also crucial and this is also evident from the results of our study. In fact, out of 55 identified training initiatives, 21 were focused on the general methodology of HTA and its application. Scientific evidence produced with the HTA approach is needed to understand the key concepts of any health technology (drugs, medical device or public health interventions) and a basic knowledge of the HTA methodology should be “at hand” of the healthcare professionals and all stakeholders of health system. Another important finding emerged from our study is the greater consideration, also in the training field, of pharmaceutics compared to other health technologies. In fact, in the courses identified the technologies considered were mainly pharmaceutics, two genetic therapies and one medical devices. Over the years, the HTA methodology was mainly applied to pharmaceutics evaluation. Taking into account the rapid developments in particular in pharmaceutical sector, such as the oncology, over the last decade, HTA was used to support mostly decision-making related to drugs, aiming to assess their therapeutic value and the economic impact on health systems (73).

However, the application of HTA will must be implemented as well as training in this field, also for medical devices and for other health technologies such as digital ones, in relation to the disruptive innovation of recent years and the near future (74). On December 2021, the new Regulation on HTA has been adopted. The Regulation on HTA (75) enters into force in January 2022 and applies as of January 2025. It contributes to improving the availability of innovative health technologies—such as medicines, certain medical devices, medical equipment, and prevention and treatment methods—for EU patients, it ensures efficient use of resources and strengthens the quality of HTA across the Union. Furthermore, it provides a transparent and inclusive framework by establishing a Coordination Group of HTA national or regional authorities, a stakeholder network and by laying down rules on the involvement in joint clinical assessments and joint scientific consultations of patients, clinical experts and other relevant experts. It will also reduce duplication of efforts for national HTA authorities and industry, facilitate business predictability and ensure the long-term sustainability of EU HTA cooperation (75).

Obviously, the application of the new Regulation on HTA further imposes training programs for healthcare professionals and for all actors of the health system in the HTA field, in order to ensure its correct application throughout the EU.

Over the years, the lack of training opportunities was considered the main challenge for the implementation of the HTA application (76, 77). The 2015 global survey of national HTA authorities reported the lack of qualified human resources, information, knowledge and methodology, as main barriers in undertaking and using HTA in nearly 59 countries (72). Despite the various efforts made by several countries, the scarcity of training programs represents a critical issue concerning the necessity to encounter training needs of healthcare professionals using the HTA approach.

HTA requires multi-disciplinary skills and core competencies, which should be synergistically involved and empowered. A recent article on capacity building in HTA agencies reported the ability to design, conduct, evaluate and to understand HTA applications to decision making, as the main training needs for an efficient and effective HTA process (78). According to the proposal by the HTAi Scientific Development and Capacity Building Committee, HTA capacity building represents a much broader suite of activities than simply training of core HTA staff in technical competencies (79).

Our study is the first that, to our knowledge, mapped the existing training initiatives in HTA for healthcare professionals, provided by international HTA agencies and organizations around the world, underlining the need for greater training in HTA for this target population. Training that should be focused, in particular, on the general HTA methodology and on all dimensions related to health technology, not just the economic one.

However, the results of the present work should be interpreted in the light of some methodological limitations. The desk research only explored the websites of EUnetHTA, INAHTA, and HTAi members, suggesting that other HTA agencies/organizations, not affiliated with these networks, might not were captured. Although the search was extensive in English, French, Portuguese, Spanish, Italian, and German, training initiatives in other national languages might not were retrieved, thus indicating a potential publication bias. Moreover, given that not all the courses were publicly available, we could not extract all their information. In this overview, it was not even possible to verify which ones were paid and which were free. However, price information could be very useful as some training courses can be very expensive and this could be an obstacle, for example, for participants from low-income countries.

However, our search strategy was extensive and conducted rigorously, providing a wide overview of the training initiatives provided by HTA agencies/organizations at European and international level.

In conclusion, considering that HTA represents a bridge between scientific evidence and policy decision-making, the training of healthcare professionals in this field should be a key driver for increasing the correct use of HTA, as an applicable tool for health governance and keeping up with technological innovations. From our work emerged the need for developing a structured HTA capacity-building, providing a basic knowledge in HTA principles and methodology, and enhancing the evaluation of all domains of the health technology assessment process. Future skills implementation programs will need to pay particular attention to the training needs of all healthcare professionals involved in the use of health technologies and in their assessment process.

## Data Availability Statement

The original contributions presented in the study are included in the article/supplementary material, further inquiries can be directed to the corresponding author.

## Author Contributions

SB and GC critically reviewed the manuscript and conceived the study critically reviewed the manuscript. IH and CC identified the HTA training initiatives through a search of websites of EUnetHTA, INAHTA and HTAi members, performed the data extraction, and contributed equally to the drafting of the paper. GC supervised IH and CC. GC, IH, and CC critically discussed and interpreted the results of the desk research. All authors contributed to the article and approved the final version.

## Funding

This work was supported by the National Center for Disease Prevention and Control (CCM), Italian Ministry of Health (CUP J54I20000350001).

## Conflict of Interest

The authors declare that the research was conducted in the absence of any commercial or financial relationships that could be construed as a potential conflict of interest.

## Publisher's Note

All claims expressed in this article are solely those of the authors and do not necessarily represent those of their affiliated organizations, or those of the publisher, the editors and the reviewers. Any product that may be evaluated in this article, or claim that may be made by its manufacturer, is not guaranteed or endorsed by the publisher.
